# Novel Insights Linking lncRNAs and Metabolism With Implications for Cardiac Regeneration

**DOI:** 10.3389/fphys.2021.586927

**Published:** 2021-03-10

**Authors:** Magda Correia, Bruno Bernardes de Jesus, Sandrina Nóbrega-Pereira

**Affiliations:** ^1^Department of Medical Sciences and Institute of Biomedicine – iBiMED, University of Aveiro, Aveiro, Portugal; ^2^Instituto de Medicina Molecular João Lobo Antunes, Faculdade de Medicina, Universidade de Lisboa, Lisbon, Portugal

**Keywords:** lncRNAs, metabolism, mitochondria, regeneration, heart

## Abstract

Heart disease is the leading cause of mortality in developed countries. The associated pathology is typically characterized by the loss of cardiomyocytes that leads, eventually, to heart failure. Although conventional treatments exist, novel regenerative procedures are warranted for improving cardiac regeneration and patients well fare. Whereas following injury the capacity for regeneration of adult mammalian heart is limited, the neonatal heart is capable of substantial regeneration but this capacity is lost at postnatal stages. Interestingly, this is accompanied by a shift in the metabolic pathways and energetic fuels preferentially used by cardiomyocytes from embryonic glucose-driven anaerobic glycolysis to adult oxidation of substrates in the mitochondria. Apart from energetic sources, metabolites are emerging as key regulators of gene expression and epigenetic programs which could impact cardiac regeneration. Long non-coding RNAs (lncRNAs) are known master regulators of cellular and organismal carbohydrate and lipid metabolism and play multifaceted functions in the cardiovascular system. Still, our understanding of the metabolic determinants and pathways that can promote cardiac regeneration in the injured hearth remains limited. Here, we will discuss the emerging concepts that provide evidence for a molecular interplay between lncRNAs and metabolic signaling in cardiovascular function and whether exploiting this axis could provide ground for improved regenerative strategies in the heart.

## Introduction

The World Health Organization (WHO) has been reporting every year that cardiovascular diseases (CVD) are the leading cause of death in the world. Although currently there are large range of pharmaceutical drugs and surgical options that prevent further deterioration or restore function to the failing heart, for end-stage heart failure, the only long-term selection is heart transplantation which presents several limitations (Hudson and Porrello, 2013). Therefore, the development of improved cardiac regenerative strategies is an area of growing interest.

Subsequent to cardiac injury, cardiomyocytes undergo necrotic and apoptotic cell death and cardiac fibroblasts are activated to produce collagen and other extracellular matrix components, leading to fibrosis and harmed cardiac function (Song et al., 2012; Hashimoto et al., 2018). The main goal of regenerative cardiovascular medicine is to repair injured hearts by replacing cardiomyocytes and diminishing fibrosis. In order to suppress the outcomes of heart failure several regenerative strategies have been proposed, including post-injury activation of cardiomyocyte proliferation, recruitment of stem cells or progenitor cells, delivery of *de novo* cardiomyocytes from iPSCs, and direct reprogramming of resident cardiac fibroblasts (CFs) into induced cardiac-like myocytes (iCLMs) (Ieda et al., 2010; Song et al., 2012; Ghiroldi et al., 2018; Hashimoto et al., 2018). Although most strategies aim at recovering cardiac function post-injury, approaches that target mechanisms of heart regeneration at pre-injury and during injury events can also potentially be conceived (Uygur and Lee, 2016; Galdos et al., 2017; Tzahor and Poss, 2017).

Besides holding great promise, most cardiac regenerative strategies still lack effective clinical outcomes (Ghiroldi et al., 2017; Hashimoto et al., 2018). Therefore understanding the molecular mechanisms and players governing cardiac regeneration in the injured hearth is warranted for improving the efficiency of cardiac regenerative strategies. In this context, long non-coding RNAs (lncRNAs), a class of > 200 nucleotides-long ribonucleic acid sequences, are abundantly expressed in the cardiovascular system and are part of a complex regulatory network governing cardiovascular function in health and disease (Bär et al., 2016; Das et al., 2018; Hobuß et al., 2019). Essential roles for some lncRNAs in heart development have been described (Matkovich et al., 2014; Bär et al., 2016; Haemmig et al., 2017) and exploring the role of lncRNAs in cardiovascular function may facilitate the development of new therapeutics for treating cardiovascular disease (Bär et al., 2016; Hobuß et al., 2019).

Although the adult mammalian heart has limited regenerative capacity, with estimation of only ≈1% *de novo* cardiomyogenesis per year (Neidig et al., 2018), the neonatal heart is capable of substantial regeneration but this capacity is lost by postnatal day (P) 7 (Soonpaa et al., 1996). Interestingly, this lost in proliferative potential is accompanied by a shift in the main energetic metabolic pathway and fuels preferentially used by cardiomyocytes from embryonic glucose-driven anaerobic glycolysis to adult oxygen-dependent oxidative phosphorylation (OXPHOS) of pyruvate and fatty acids (FAs) in the mitochondria (Lopaschuk et al., 1992; Lehman and Kelly, 2002). Apart from energetic sources, metabolites are key regulators of gene expression programs by acting as essential substrates or cofactors for chromatin-modifying enzymes (Intlekofer and Finley, 2019). Interestingly, lncRNAs are emerging as master regulators of cellular and systemic carbohydrate and lipid metabolism with clear implications for cardiovascular function (Zhao, 2015; van Soligen, 2018; Mongelli et al., 2019), opening the possibility for a two-way communication between metabolism and lncRNAs in cardiac heart regeneration.

Here, we will discuss the emerging insights in the molecular interplay between lncRNAs and metabolism in the heart highlighting evidence for the impact of metabolic signaling in cardiac regeneration. Also, particular emphasis will be given to those lncRNAs regulating metabolic targets in the cardiovascular system and the potential modulation of the lncRNAs-metabolic axis for the development of innovative regenerative strategies.

## Can an Embryonic-Like Metabolic Program Promote Heart Regeneration?

The fetal heart’s environment is low in oxygen and FAs, thus fetal cardiomyocytes are highly dependent on glycolysis for ATP production (Lopaschuk et al., 1992). The heart suffers a major metabolic alteration driven by the physiological changes at postnatal stages, as enhanced workload and the demand for growth, that cannot be supported by glucose and lactate metabolism (Malandraki-Miller et al., 2018). The mammalian heart has to contract constantly thus, the need for an optimal energy fuel is imperative. During the early postnatal period, the number of mitochondria in cardiomyocytes increases dramatically (Mayor and Cuezva, 1985; Attardi and Schatz, 1988). Mitochondria is the organelle that coordinates the energy transduction function and it is responsible to produce more than 95% of ATP utilized by the heart (Doenst et al., 2013). Additionally, mitochondria regulates intracellular calcium homeostasis, signaling and apoptosis (Kolwicz et al., 2013). As a result, mammalian cardiomyocytes undergo extensive metabolic remodeling after birth. In order to adapt to the high-energetic demands of the postnatal life, cardiomyocytes suffer a metabolic switch and produce their energy via mitochondrial OXPHOS, a more efficient process than glycolysis (Lehman and Kelly, 2002; Vivien et al., 2016). Postnatal cardiomyocytes also revealed a shift in the energetic substrate utilization from pyruvate to FAs that are energetically more favorable (Lopaschuk et al., 1992; Lehman and Kelly, 2002). As the neonatal mammalian heart regenerative capacity is lost by P7, which corresponds with cardiomyocyte binucleation and cell-cycle arrest (Soonpaa et al., 1996; Porrello et al., 2011), one could hypothesize that the “fetal metabolic shift” would have a role in suppressing cardiomyocyte proliferation and heart repair (Martik, 2020). Currently many studies are focusing in understanding the role of mitochondrial metabolism in regulating cell-cycle arrest in postnatal cardiomyocytes with future implications in regenerative strategies.

Heart regeneration in zebrafish is incredibly effective and relies on the proliferation of pre-existing cardiomyocytes. Apart from cardiomyocytes, other cell types (such as epicardial, endocardial, immune cells and fibroblasts) respond to the heart injury and contribute for the healing process (Vivien et al., 2016; Honkoop et al., 2019). Cardiomyocytes from highly regenerative species such as zebrafish have a preference for glycolysis and increase OXPHOS activity promotes cardiomyocyte maturation and reduces the proliferative capacity (Vivien et al., 2016; Honkoop et al., 2019; Fukuda et al., 2019). Although the “fetal switch” to mitochondrial respiration has been associated to loss of the regenerative potential (Malandraki-Miller et al., 2018), the role of bioenergetics in regulating cardiogenesis remains unclear. Recent evidence suggest that hypoxia inducible factor 1 (HIF1) signaling, an important inducer of aerobic glycolysis and the Warburg effect in cancer cells (Kroemer and Pouyssegur, 2008), controls the embryonic switch toward oxidative metabolism in the developing mouse heart (Menendez-Montes et al., 2016). In midgestational mouse heart, the compact myocardium downregulates HIF1α and switches toward oxidative metabolism. Deletion of the E3 ubiquitin ligase von Hippel-Lindau (VHL) results in HIF1α hyperactivation, blocking the midgestational metabolic shift and impairing cardiac maturation and function (Menendez-Montes et al., 2016). This study highlights the VHL-HIF-mediated metabolic program as an important axis for myocardial differentiation and its potential relevance for cardiac regeneration.

The adult mammalian heart cannot regenerate lost or damaged myocardium although it does present a limited myocyte turnover that reveals insufficient for restoring contractile dysfunction. The brief window of regenerative response following injury seems to be also driven by proliferation of pre-existing cardiomyocytes (Porrello et al., 2011; Elhelaly et al., 2019). Strikingly, increase production of mitochondrial-derived reactive oxygen species (ROS) and DNA oxidation leads to cell-cycle arrest in mouse postnatal cardiomyocytes through the activation of DNA damage response pathways (Puente et al., 2014). FAs oxidation is directly linked to high ROS production and cardiomyocyte cell-cycle arrest (Cardoso et al., 2020). Moreover, the constant use of FAs as an energetic fuel provokes a dependency on this substrate as the acetyl-CoA produced from FAs oxidation inhibits the mitochondrial enzyme pyruvate dehydrogenase (PDH) and therefore the reliance on glucose and its metabolites for energy (Rindler et al., 2013). Still, whether modulating substrate utilization can directly impact DNA damage and promote cardiomyocytes cell-cycle re-entry needs further clarification. Supplementation of FAs-depleted diets to mice prolongs the postnatal window for cardiomyocyte proliferation; however, it is associated with a marked hepatomegaly and steatosis due to a compensatory increase in hepatic *de novo* fatty-acid synthesis (Cardoso et al., 2020). Moreover, deletion of the dehydrogenase kinase isoform 4 (PDK4) in adult cardiomyocytes, the main enzyme responsible for PDH inhibition and FAs usage, results in a marked shift in myocardial substrate utilization with decrease FAs and enhanced pyruvate-driven glucose oxidation, resulting in less DNA damage and increased cardiomyocyte proliferation (Cardoso et al., 2020). Pharmacological activation of PDH through administration of dichloroacetate in mice also resulted in improved glucose utilization and cardioprotective features (Cardoso et al., 2020).

In sum, recent studies are beginning to dissect the intricate relationship between the “fetal metabolic switch” and loss of cardiomyocyte proliferation where several molecular axis (as HIF signaling, ROS and bioenergetic fuels) are emerging as key regulators. This raises important questions and opportunities in the field. For instance, cardiac regenerative strategies based on the generation of induced cardiac-like myocytes (iCLMs) from iPSCs or resident cardiac fibroblasts (CFs) (Hashimoto et al., 2018), could be improved by metabolic modulation and induction of the “fetal switch”. Moreover, systemic metabolic shifts, as nutritional stages and diets, may impact cardiac regeneration post-injury in the mammalian heart (Malandraki-Miller et al., 2018). Further investigation in this field is warranted and may provide unique opportunities to boost cardiac regeneration and repair.

## LncRNAs Controlling Metabolic Pathways in the Heart

LncRNAs represent one of the most prominent but least understood transcriptome in the heart. Thousands of lncRNAs have been identified to be dynamically transcribed during development, differentiation, and maturation of cardiac myocytes (Devaux et al., 2015; He et al., 2016; Li et al., 2017; Beermann et al., 2018). Due to their unique regulatory action and tissue-specific expression, lncRNAs are attractive candidates for diagnosis of cardiovascular pathologies and regenerative strategies using several lncRNA-based therapeutic approaches (Bär et al., 2016; Bernardes de Jesus et al., 2018; Hobuß et al., 2019). lncRNAs are localized in the nucleus or the cytoplasm where they may regulate gene expression at transcriptional or posttranscriptional level, respectively, through diverse mechanisms; including epigenetic remodeling, transcriptional activation or repression (signal, decoy, guide, scaffold, or enhancer lncRNAs), formation and maintenance of sub-nuclear domains, posttranscriptional regulation and modulation of protein activity (Schonrock et al., 2012; Kornfeld and Brüning, 2014; Devaux et al., 2015; Thum and Condorelli, 2015; Muret et al., 2019).

LncRNAs are emerging as master regulators of cellular and organismal carbohydrate and lipid metabolism in adipose tissue and liver (Kornfeld and Brüning, 2014; Zhao, 2015; van Soligen, 2018; Mongelli et al., 2019; Muret et al., 2019). Alteration in serum lipid levels is one of the most relevant risk factor for CVD (Doggen et al., 2004). In the recent years, several studies have highlighted the complex contribution of lncRNAs in controlling systemic and cell-type-specific cholesterol, FAs, and triglyceride metabolism, with important implications for CVD. For instance, several lncRNAs, including *H19*, lncRNA HCV regulated 1 (*lncHR1*), *MALAT-1* and *lncARSR*, have been shown to regulate the expression of the sterol regulatory element binding protein 1c (*SREBP-1c*), a transcription factor that regulates lipid synthesis and uptake in the liver (Yan et al., 2016; Li et al., 2018; Liu et al., 2018; Zhang et al., 2018). Other examples are the liver-specific triglyceride regulator lncRNA Lancaster (*lncLSTR*) that regulates triglyceride plasma levels and energy metabolism (Li et al., 2015) and *AT102202* that inhibits cholesterol synthesis in the liver by targeting the rate limiting enzyme HMGCR (Liu et al., 2015). Whether lncRNAs-mediated control of systemic lipid metabolism has a direct impact in cardiac regeneration remains to be addressed.

As previously discussed, of particular interest are the lncRNAs controlling the “fetal metabolic switch” from embryonic glycolysis to adult mitochondrial respiration and the preferred usage of FAs as energetic fuel in differentiated cardiomyocytes. Although most of our knowledge in lncRNAs control of metabolism comes from studies in lipogenic tissues and/or cancer energetics (Gomes et al., 2019), some mechanistic insights in cardiac muscle development and function, particularly concerning mitochondrial metabolism, are beginning to arise (Table 1). Due to the implication of mitochondrial-dependent FAs oxidation and ROS production in the loss of cardiomyocyte proliferation (Puente et al., 2014; Cardoso et al., 2020), lncRNAs regulating these pathways are particularly attractive for cardiac regeneration.

**TABLE 1:** LncRNAs with characterized and/or potential metabolic targets in the heart.

LncRNA	Tissue/Cell type	Species	Mechanism of action	Target genes	Metabolic impact	References
*LINC00116*	Heart and skeletal muscle	Human, mouse	Mitochondrial-related sORFs	Mtln	Mtln enhances mitochondrial respiration and fatty acid β-oxidation	Makarewich et al., 2018; Stein et al., 2018
*NEAT1*	Heart and skeletal muscle, HeLa cells	Human, mouse	Establishment of paraspeckles	*CYCS*, *NDUFA13, CPT1A*	Induces dysfunction of mitochondrial respiration and fission	Wang et al., 2018, 2019
*CARL*	Heart, cardiomyocytes	Mouse	Decoy, sequester microRNA-539	PHB2	Suppress mitochondrial fission and apoptosis	Wang et al., 2014
*CDKN2B-AS1 (ANRIL)*	HEK, HeLa cells	Human	Scaffold	*ADIPOR1, TMEM258, VAMP3*	Impacts glucose and fatty acid metabolism	Bochenek et al., 2013
*MT-LIPCAR*	Plasma	Human	Unknown	*CYTB, COX2 (?)*	Unknown	Kumarswamy et al., 2014
*H19*	Heart and neonatal cardiomyocytes	Human, mouse, rat	MicroRNA-675 precursor	*VDAC1*, *DIRAS3*	Decreases oxidative stress, apoptosis and autophagy	Li et al., 2016; Zhuo et al., 2017

*sORFs, small opening reading frames; Mitoregulin, Mtln; NEAT1, nuclear enriched abundant transcript 1; CYCS, cytochrome c; NDUFA13, NADH:Ubiquinone Oxidoreductase Subunit A13; CPT1A, Carnitine Palmitoyltransferase 1A; CARL, cardiac apoptosis-related lncRNA; PHB2, Prohibitin 2; ANRIL, antisense non-coding RNA in the INK4 locus; HEK, human embryonic kidney; ADIPOR1, adiponectin receptor 1; TMEM258, Transmembrane Protein 258; VAMP3, vesicle associated membrane protein 3; MT-LIPCAR, Mitochondrially Encoded Long Non-Coding Cardiac Associated RNA; CYTB, Cytochrome B; COX2, Cytochrome C Oxidase II; VDAC1, voltage-dependent anion channel 1; DIRAS3, DIRAS Family GTPase 3.*

In heart and skeletal muscle aged tissue, the lncRNA *LINC00116* is among the most significantly downregulated gene (GEO: GSE362 and GSE674). Interestingly, a small region of the most predominant isoform is actively translated in human and mouse muscle and has been found to encode a highly conserved transmembrane microprotein, named mitoregulin (Mtln), also known as Micropeptide regulator of β-oxidation (MOXI), where it associates with the mitochondrial trifunctional protein (MTP), an enzyme complex that plays a critical role in fatty acid β-oxidation (Makarewich et al., 2018; Stein et al., 2018). Isolated heart and skeletal muscle mitochondria from MOXI knockout mice preferentially oxidize carbohydrates over fatty acids, while transgenic MOXI overexpression leads to enhanced β-oxidation. MOXI knockout mice also exhibit a profound reduction in exercise capacity, highlighting the role of MOXI in metabolic control (Makarewich et al., 2018). The impact of Mtln expression in cardiovascular disease and regeneration is still unclear but GTEx portal annotates the existence of common genetic variants that strongly associate with *LINC00116* expression in the human heart (Stein et al., 2018). *NEAT1* (nuclear enriched abundant transcript 1) is another lncRNA with increase expression in non-regenerative cardiomyocytes (Table 1). In skeletal muscle, *NEAT1* modulates myogenesis by accelerating myoblast proliferation and suppressing myoblast differentiation and fusion (Wang et al., 2019). *NEAT1* act by recruiting EZH2 to target gene promoters, decreasing the expression of the cyclin-dependent kinase inhibitor *p21* and suppressing the myoblast differentiation program. Strikingly, several mitochondrial regulators have been identified to associate to *NEAT1* in paraspeckles, a type of nuclear body with multiple roles in gene expression (Wang et al., 2018). Specifically, *NEAT1* depletion lead to profound effects on mitochondrial dynamics and function by altering the paraspeckles-specific sequestration of essential mito-mRNAs, including *CYCS* (cytochrome c), *NDUFA13* (NADH:Ubiquinone Oxidoreductase Subunit A13) and *CPT1A* (Carnitine Palmitoyltransferase 1A) (Wang et al., 2018) and *NEAT1*-depleted HeLa cells show reduced mitochondrial DNA, ATP production and proliferation rate (Wang et al., 2018).

Cardiac muscle is an extremely metabolically active tissue that undergoes significant changes in energy metabolism in disease. In mouse cardiomyocytes, cardiac apoptosis-related lncRNA (*CARL*) binds to and sequester microRNA-539, a microRNA found to target the mRNA of the PHB2 sub-unit of prohibitin, a protein localized to the inner mitochondrial membrane that regulates mitochondrial homeostasis (Wang et al., 2014). Downregulation of PHB2 during pathological insults was found to be dependent on upregulation of microRNA-539. *CARL* acts as the endogenous sponge for this microRNA suppressing mitochondrial fission and cardiomyocyte apoptosis (Wang et al., 2014), highlighting the therapeutic potential of modulating lncRNAs during myocardial infarction. The lncRNA *CDKN2B−AS1* (also known as *ANRIL*) has been described as a genetic risk factor for coronary artery disease (CAD) (Deloukas et al., 2013). *ANRIL* expression level is associated with left ventricular dysfunction after myocardial infarction (Vausort et al., 2014). Experimental manipulation in several human cell lines (including HEK and HeLa), revealed that *ANRIL* knock-down decreases the expression of *ADIPOR1* (adiponectin receptor 1), *TMEM258* (also known as *C11ORF10* for chromosome 11 open reading frame 10) and *VAMP3* (vesicle associated membrane protein 3), both at the transcript and protein level, which are important genes in the regulation of glucose and fatty-acid metabolism (Bochenek et al., 2013). However, the impact of *ANRIL*-mediated metabolic regulation in cardiomyocytes remains to be explored. Conversely, in patients with myocardial infarction the levels of the lncRNA hypoxia inducible factor 1A antisense RNA 2 *(HIF1A-AS2)* was found to be upregulated (Vausort et al., 2014). In humans, the HIF pathway is induced early in acute myocardial and remains activated in chronic human heart failure (Zolk et al., 2008). Due to the role of the HIF signaling in controlling myocardial metabolism and differentiation in the neonatal heart (Menendez-Montes et al., 2016) and the implication of the lncRNA *lincRNA-p21* in hypoxia-enhanced glycolysis (Yang et al., 2014), manipulation of the lncRNA/HIF regulatory network might constitute an attractive target to modulate cardiac regeneration.

Type 2 diabetes (T2D) is a multifactorial disorder characterized, among other aspects, by high blood glucose and lipid levels (hyperglycemia and hyperlipidemia) in association with insulin resistance and atherosclerosis (Bornfeldt and Tabas, 2011) and diabetic cardiomyopathy (DCM) is a critical complication of T2D (Jia et al., 2018). Studies suggest that lncRNAs that regulate metabolic targets are aberrantly regulated in DCM, thus targeting lncRNAs could have potential implications for DCM diagnosis and therapy. The mitochondrial long intergenic non-coding RNA predicting cardiac remodeling (*MT-LIPCAR*) is a lncRNA possibly transcribed from mitochondrial DNA that cross the membrane barrier being released into circulation (Dorn, 2014). Plasma levels of *MT-LIPCAR* were positively associated with left ventricular diastolic dysfunction in T2D patients with DCM showing prognostic value as an indicator of heart failure and patient mortality. *MT-LIPCAR* was the first proof that plasma lncRNAs might be used for cardiovascular disease prognostic (Kumarswamy et al., 2014). Despite the invaluable potential as a cardiac biomarker, *MT-LIPCAR* targets and metabolic impact remains unclear. Evidence suggest that the complete *MT-LIPCAR* sequence could map to the mitochondrial genes *CYTB* (Mitochondrially Encoded Cytochrome B) and *COX2* (Mitochondrially Encoded Cytochrome C Oxidase II) (Dorn, 2014), raising further questions regarding MT-LIPCAR biogenesis as a mitochondrial or nuclear pseudogene transcript. *H19* is a lncRNA transcribed from H19/insulin-like growth factor-II (IGF2) genomic imprinted cluster which accumulates in cardiomyocytes of the mature myocardium in humans and rodents (Pant et al., 2018; Viereck et al., 2020). Decrease expression of cardiac *H19* was reported in a rat model of DCM (Li et al., 2016; Zhuo et al., 2017). Overexpression of *H19* in myocardial tissue was able to suppress oxidative stress, inflammation and improve left ventricular function leading to DCM amelioration. Mechanistically, *H19* serves as template for microRNA-675 expression from its first exon (Zhang et al., 2017; Pant et al., 2018). Since microRNA-675 has multiple biological targets, *H19* is able to regulate a number of mitochondrial functions including suppression of apoptosis by targeting voltage-dependent anion channel 1 (*VDAC1*) (Li et al., 2016), and inhibiting autophagy in cardiomyocytes exposed to high glucose through down-regulation of the GTP- binding protein Di-Ras-3 (*DIRAS3*) (Zhuo et al., 2017).

In sum, recent work on lncRNAs has started to shed light on their regulatory potential in controlling heart metabolism in health and disease, opening the possibility to explore lncRNAs-mediated metabolic control as a strategy to improve cardiac regeneration and heart function.

## LncRNAs and Metabolites as Central Epigenetic Players in Gene Expression Regulation

An hallmark function of lncRNAs is their ability to mediate epigenetic regulation and in the heart, lncRNAs have crucial roles in regulating cardiac chromatin structure during development and pathological remodeling (Schonrock et al., 2012). lncRNAs exhibit tissue-specific regulated expression patterns which are frequently lost during disease (Cabili et al., 2011). However, the regulation of lncRNAs expression during different stages of cardiac development and in disease is still under investigation. Strikingly, inhibition of epigenetic modifications was shown to impact the expression pattern of lncRNAs (Schonrock et al., 2012). Metabolites are emerging as key regulators of gene expression programs and epigenetic modifications, acting as essential substrates or cofactors for enzymes that deposit or remove chemical modifications in DNA and/or histones (Intlekofer and Finley, 2019). FAs and cholesterol have been shown to regulate lncRNAs expression in lipogenic tissues placing metabolism as a central regulator of epigenetic-driven lncRNAs transcription. For instance, the expression of the lncRNAs *H19* and *MALAT1* is upregulated by FAs exposure, coinciding with an increase in (SREBP)-1c in hepatic cells (van Soligen, 2018) and *HULC* is induced by cholesterol in hepatoma cells via the retinoic receptor RXRA, leading to lipogenesis (Cui et al., 2015). Recent evidence suggests that lipid metabolism also impact lncRNAs expression in the cardiovascular system. For instance, the lncRNA *CHROME, a* master regulator of cholesterol homeostasis, is upregulated in atherosclerotic vascular disease in non-human primates and conversely, *CHROME* expression is influenced by dietary and cellular cholesterol (Hennessy et al., 2019). Although evidence for the direct implication of nutritional signals in the epigenetic alterations that govern lncRNAs expression in the heart is still at its early days, it seems clear that lncRNAs and metabolic signaling can engage in a two-way communication road in the control of gene expression that impacts cellular and systemic metabolism. Moreover, nutritional cues have been shown to control the specification of skeletal cell fate, highlighting the possibility for a similar network to take place in cardiomyocyte progenitors. When lipids are scarce, skeletal muscle progenitors activate the expression of forkhead box O (FOXO) transcription factors leading to a Sox9-dependent suppression of FAs oxidation and chondrogenic commitment (van Gastel et al., 2020). Moreover, glucose metabolism is crucial for muscle stem cells (MuSCs) commitment. In proliferating MuSCs, glucose is dispensable for mitochondrial respiration and becomes available for maintaining high histone acetylation via acetyl-CoA, whereas differentiating MuSCs increases glucose oxidation and has consequently reduced acetylation (Yucel et al., 2019). PDH is pivotal for this switch and determines the differentiation potential of myogenic progenitors during muscle regeneration (Yucel et al., 2019). Whether metabolic fuels also directly impinge cardiomyocyte cell fate decisions and dietary cues can modulate cardiac regeneration (for instance, by controlling lncRNAs expression) are exciting possibilities that require further investigation.

## Concluding Remarks

Given the emerging regulatory potential of lncRNAs, it is undoubted that these molecules offer potential solutions in the pursuit for cardiac regeneration (Hudson and Porrello, 2013). In the recent years, several lncRNAs with characterized and/or potential metabolic targets in the heart have been identified (Table 1) and a link between metabolic pathways and cardiac proliferative potential has been established. But can we boost cardiac regeneration by modulating the lncRNAs-metabolic axis? Emerging evidence suggests that exploring the two-way communication road between lncRNAs and cardiac (or systemic) metabolism may offer new perspectives and opportunities for increasing the regenerative potential of the injured heart.

## Author Contributions

MC, BB, and SN-P planned, wrote, and discussed the manuscript. BB and SN-P revised the manuscript. All authors contributed to the article and approved the submitted version.

## Conflict of Interest

The authors declare that the research was conducted in the absence of any commercial or financial relationships that could be construed as a potential conflict of interest.

## References

[B1] Attardi G. Schatz G. (1988). Biogenesis of mitochondria. *Annu. Rev. Cell Biol.* 4 289–333. 10.1146/annurev.cb.04.110188.001445 2461720

[B2] Bär C. Chatterjee S. Thum T. (2016). Long noncoding RNAs in cardiovascular pathology. *Diagn. Ther.Circ.* 134 1484–1499. 10.1161/CIRCULATIONAHA.116.023686 27821419

[B3] Beermann J. Kirste D. Iwanov K. Lu D. Kleemiß F. Kumarswamy R. (2018). A large shRNA library approach identifies lncRNA Ntep as an essential regulator of cell proliferation. *Cell Death Differ.* 25 307–318. 10.1038/cdd.2017.158 29099486 PMC5762845

[B4] Bernardes de Jesus B. Marinho S. P. Barros S. Sousa-Franco A. Alves-Vale C. Carvalho T. (2018). Silencing of the lncRNA Zeb2-NAT facilitates reprogramming of aged fibroblasts and safeguards stem cell pluripotency. *Nat. Commun.* 9:94. 10.1038/s41467-017-01921-6 29311544 PMC5758807

[B5] Bochenek G. Häsler R. Mokhtari N. E. El König I. R. Loos B. G. Jepsen S. (2013). The large non-coding RNA ANRIL, which is associated with atherosclerosis, periodontitis and several forms of cancer, regulates ADIPOR1, VAMP3 and C11ORF10. *Hum. Mol. Genet.* 22 4516–4527. 10.1093/hmg/ddt299 23813974

[B6] Bornfeldt K. E. Tabas I. (2011). Insulin resistance, hyperglycemia, and atherosclerosis. *Cell Metab.* 14 575–585. 10.1016/j.cmet.2011.07.015 22055501 PMC3217209

[B7] Cabili M. Trapnell C. Goff L. Koziol M. Tazon-Vega B. Regev A. (2011). Integrative annotation of human large intergenic noncoding RNAs reveals global properties and specific subclasses. *Genes Dev.* 25 1915–1927. 10.1101/gad.17446611 21890647 PMC3185964

[B8] Cardoso A. C. Lam N. T. Savla J. J. Nakada Y. Pereira A. H. M. Elnwasany A. (2020). Mitochondrial substrate utilization regulates cardiomyocyte cell-cycle progression. *Nat. Metab.* 2 167–178. 10.1038/s42255-020-0169-x 32617517 PMC7331943

[B9] Cui M. Xiao Z. Wang Y. Zheng M. Song T. Cai X. (2015). Long noncoding RNA HULC modulates abnormal lipid metabolism in hepatoma cells through an miR-9-mediated RXRA signaling pathway. *Cancer Res.* 75 846–857. 10.1158/0008-5472.CAN-14-1192 25592151

[B10] Das A. Samidurai A. Salloum F. N. (2018). Deciphering non-coding RNAs in cardiovascular health and disease. *Front. Cardiovasc. Med.* 5:73. 10.3389/fcvm.2018.00073 30013975 PMC6036139

[B11] Deloukas P. Kanoni S. Willenborg C. Farrall M. Assimes T. L. Thompson J. R. (2013). Large-scale association analysis identifies new risk loci for coronary artery disease. *Nat. Genet.* 45 25–33. 10.1038/ng.2480 23202125 PMC3679547

[B12] Devaux Y. Zangrando J. Schroen B. Creemers E. E. Pedrazzini T. Chang C. P. (2015). Long noncoding RNAs in cardiac development and ageing. *Nat. Rev. Cardiol.* 12 415–425. 10.1038/nrcardio.2015.55 25855606

[B13] Doenst T. Nguyen T. D. Abel E. D. (2013). Cardiac metabolism in heart failure. *Circ. Res.* 113 709–724. 10.1161/circresaha.113.300376 23989714 PMC3896379

[B14] Doggen C. J. M. Smith N. L. Lemaitre R. N. Heckbert S. R. Rosendaal F. R. Psaty B. M. (2004). Serum lipid levels and the risk of venous thrombosis. *Arterioscler. Thromb. Vasc. Biol.* 24 1970–1975. 10.1161/01.ATV.0000143134.87051.46 15331431

[B15] Dorn G. W. II (2014). LIPCAR: a mitochondrial lnc in the noncoding RNA chain? *Circ. Res.* 114 1548–1550. 10.1161/CIRCRESAHA.114.304028 24812345 PMC4048953

[B16] Elhelaly W. M. Cardoso A. C. Pereira A. H. M. Elnawasany A. Ebrahimi S. Nakada Y. (2019). C-Kit cells do not significantly contribute to cardiomyogenesis during neonatal heart regeneration. *Circulation* 139 559–561. 10.1161/CIRCULATIONAHA.117.033150 30664377 PMC6389270

[B17] Fukuda R. Aharonov A. Ong Y. T. Stone O. A. El-Brolosy M. A. Maischein H. M. (2019). Metabolic modulation regulates cardiac wall morphogenesis in zebrafish. *eLife* 8 1–14. 10.7554/eLife.50161 31868165 PMC7000217

[B18] Galdos F. X. Guo Y. Paige S. L. Vandusen N. J. Wu S. M. Pu W. T. (2017). Cardiac regeneration: lessons from development. *Circ. Res.* 120 941–959. 10.1161/CIRCRESAHA.116.309040 28302741 PMC5358810

[B19] Ghiroldi A. Piccoli M. Ciconte G. Pappone C. Anastasia L. (2017). Regenerating the human heart: direct reprogramming strategies and their current limitations. *Basic Res. Cardiol.* 112 1–14. 10.1007/s00395-017-0655-9 29079873

[B20] Ghiroldi A. Piccoli M. Cirillo F. Monasky M. M. Ciconte G. Pappone C. (2018). Cell-Based therapies for cardiac regeneration: a comprehensive review of past and ongoing strategies. *Int. J. Mol. Sci.* 19:3194. 10.3390/ijms19103194 30332812 PMC6214096

[B21] Gomes C. P. Nóbrega-Pereira S. Domingues-Silva B. Rebelo K. Alves-Vale C. Marinho S. P. (2019). An antisense transcript mediates MALAT1 response in human breast cancer. *BMC Cancer* 19:771. 10.1186/s12885-019-5962-0 31382922 PMC6683341

[B22] Haemmig S. Simion V. Yang D. Deng Y. Feinberg M. W. (2017). Long noncoding RNAs in cardiovascular disease, diagnosis, and therapy. *Curr. Opin. Cardiol.* 32 776–783. 10.1097/HCO.0000000000000454 28786864 PMC5892448

[B23] Hashimoto H. Olson E. N. Bassel-Duby R. (2018). Therapeutic approaches for cardiac regeneration and repair. *Nat. Rev. Cardiol.* 15 585–600. 10.1038/s41569-018-0036-6 29872165 PMC6241533

[B24] He C. Hu H. Wilson K. D. Wu H. Feng J. Xia S. (2016). Systematic characterization of long noncoding RNAs reveals the contrasting coordination of cis- and trans-molecular regulation in human fetal and adult hearts. *Circ. Cardiovasc. Genet.* 9 110–118. 10.1161/CIRCGENETICS.115.001264 26896382 PMC4862831

[B25] Hennessy E. J. van Solingen C. Scacalossi K. R. Ouimet M. Afonso M. S. Prins J. (2019). The long noncoding RNA CHROME regulates cholesterol homeostasis in primates. *Nat. Metab.* 1 98–110. 10.1038/s42255-018-0004-9 31410392 PMC6691505

[B26] Hobuß L. Bär C. Thum T. (2019). Long non-coding RNAs: at the heart of cardiac dysfunction? *Front. Physiol.* 10:30. 10.3389/fphys.2019.00030 30761015 PMC6361744

[B27] Honkoop H. de Bakker D. E. M. Aharonov A. Kruse F. Shakked A. Nguyen P. D. (2019). Single-cell analysis uncovers that metabolic reprogramming by ErbB2 signaling is essential for cardiomyocyte proliferation in the regenerating heart. *eLife* 8 1–27. 10.7554/eLife.50163 31868166 PMC7000220

[B28] Hudson J. E. Porrello E. R. (2013). The non-coding road towards cardiac regeneration. *J Cardiovasc Transl Res.* 6 909–923. 10.1007/s12265-013-9486-8 23797382

[B29] Ieda M. Fu J. Delgado-olguin P. Vedantham V. Hayashi Y. Bruneau B. G. (2010). Direct reprogramming of fibroblasts into functional cardiomyocytes by defined factors. *Cell* 142 375–386. 10.1016/j.cell.2010.07.002 20691899 PMC2919844

[B30] Intlekofer A. M. Finley L. W. S. (2019). Metabolic signatures of cancer cells and stem cells. *Nat. Metab.* 1 177–188. 10.1038/s42255-019-0032-0 31245788 PMC6594714

[B31] Jia G. Hill M. A. Sowers J. R. (2018). Diabetic cardiomyopathy: an update of mechanisms contributing to this clinical entity. *Circ. Res.* 122 624–638. 10.1161/CIRCRESAHA.117.311586 29449364 PMC5819359

[B32] Kolwicz S. C. Purohit S. Tian R. (2013). Cardiac metabolism and its interactions with contraction, growth, and survival of cardiomyocytes. *Circ. Res.* 113 603–616. 10.1161/CIRCRESAHA.113.302095 23948585 PMC3845521

[B33] Kornfeld J. W. Brüning J. C. (2014). Regulation of metabolism by long, non-coding RNAs. *Front. Genet.* 5:57. 10.3389/fgene.2014.00057 24723937 PMC3971185

[B34] Kroemer G. Pouyssegur J. (2008). Tumor cell metabolism: cancer’s achilles’. *Heel. Cancer Cell* 13 472–482. 10.1016/j.ccr.2008.05.005 18538731

[B35] Kumarswamy R. Bauters C. Volkmann I. Maury F. Fetisch J. Holzmann A. (2014). Circulating long noncoding RNA, LIPCAR, predicts survival in patients with heart failure. *Circ. Res.* 114 1569–1575. 10.1161/CIRCRESAHA.114.303915 24663402

[B36] Lehman J. J. Kelly D. P. (2002). Transcriptional activation of energy metabolic switches in the developing and hypertrophied heart. *Clin. Exp. Pharmacol. Physiol.* 29 339–345. 10.1046/j.1440-1681.2002.03655.x 11985547

[B37] Li D. Guo L. Deng B. Li M. Yang T. Yang F. (2018). Long non-coding RNA HR1 participates in the expression of SREBP-1c through phosphorylation of the PDK1/AKT/FoxO1 pathway. *Mol. Med. Rep.* 18 2850–2856. 10.3892/mmr.2018.9278 30015961 PMC6102743

[B38] Li P. Ruan X. Yang L. Kiesewetter K. Zhao Y. Luo H. (2015). A liver-enriched long non-coding RNA, lncLSTR, regulates systemic lipid metabolism in mice. *Cell Metab.* 21 455–467. 10.1016/j.cmet.2015.02.004 25738460 PMC4350020

[B39] Li X. Wang H. Yao B. Xu W. Chen J. Zhou X. (2016). lncRNA H19/miR-675 axis regulates cardiomyocyte apoptosis by targeting VDAC1 in diabetic cardiomyopathy. *Sci. Rep.* 6:36340. 10.1038/srep36340 27796346 PMC5087087

[B40] Li Y. Zhang J. Huo C. Ding N. Li J. Xiao J. (2017). Dynamic organization of lncRNA and Circular RNA regulators collectively controlled cardiac differentiation in humans. *EBioMedicine* 24 137–146. 10.1016/j.ebiom.2017.09.015 29037607 PMC5652025

[B41] Liu C. Yang Z. Wu J. Zhang L. Lee S. Shin D. -J. (2018). Long noncoding RNA H19 interacts with polypyrimidine tract-binding protein 1 to reprogram hepatic lipid homeostasis. *Hepatology.* 67 1768–1783. 10.1002/hep.29654 29140550 PMC5906152

[B42] Liu G. Zheng X. Xu Y. Lu J. Chen J. Huang X. (2015). Long non-coding RNAs expression profile in HepG2 cells reveals the potential role of long non-coding RNAs in the cholesterol metabolism. *Chin. Med. J. (Engl).* 128 91–97. 10.4103/0366-6999.147824 25563320 PMC4837827

[B43] Lopaschuk G. D. Collins-Nakai R. L. Itoi T. (1992). Developmental changes in energy substrate use by the heart. *Cardiovasc. Res.* 26 1172–1180. 10.1093/cvr/26.12.1172 1288863

[B44] Makarewich C. A. Baskin K. K. Munir A. Z. Bezprozvannaya S. Sharma G. Khemtong C. (2018). MOXI Is a mitochondrial micropeptide that enhances fatty Acid β-Oxidation. *Cell Rep.* 23 3701–3709. 10.1016/j.celrep.2018.05.058 29949755 PMC6066340

[B45] Malandraki-Miller S. Lopez C. A. Al-Siddiqi H. Carr C. A. (2018). Changing metabolism in differentiating cardiac progenitor cells—can stem cells become metabolically flexible cardiomyocytes? *Front. Cardiovasc. Med.* 5:119. 10.3389/fcvm.2018.00119 30283788 PMC6157401

[B46] Martik M. L. (2020). Metabolism makes and mends the heart. *eLife* 9 9–11. 10.7554/elife.54665 32014108 PMC7000214

[B47] Matkovich S. J. Edwards J. R. Grossenheider T. C. De Guzman Strong C. Dorn G. W. (2014). Epigenetic coordination of embryonic heart transcription by dynamically regulated long noncoding RNAs. *Proc. Natl. Acad. Sci. U.S.A.* 111 12264–12269. 10.1073/pnas.1410622111 25071214 PMC4143054

[B48] Mayor F. Cuezva J. M. (1985). Hormonal and metabolic changes in the perinatal period. *Biol. Neonate* 48 185–196. 10.1159/000242171 2998491

[B49] Menendez-Montes I. Escobar B. Palacios B. Gómez M. J. Izquierdo-Garcia J. L. Flores L. (2016). Myocardial VHL-HIF signaling controls an embryonic metabolic switch essential for cardiac maturation. *Dev. Cell* 39 724–739. 10.1016/j.devcel.2016.11.012 27997827

[B50] Mongelli A. Martelli F. Farsetti A. Gaetano C. (2019). The dark that matters: long noncoding RNAs as master regulators of cellular metabolism in noncommunicable diseases. *Front. Physiol.* 10:369. 10.3389/fphys.2019.00369 31191327 PMC6539782

[B51] Muret K. Désert C. Lagoutte L. Boutin M. Gondret F. Zerjal T. (2019). Long noncoding RNAs in lipid metabolism: literature review and conservation analysis across species. *BMC Genomics* 20:882. 10.1186/s12864-019-6093-3 31752679 PMC6868825

[B52] Neidig L. E. Weinberger F. Palpant N. J. Mignone J. Martinson A. M. Sorensen D. W. (2018). Evidence for minimal cardiogenic potential of stem cell antigen 1-positive cells in the adult mouse heart. *Circulation* 138 2960–2962. 10.1161/CIRCULATIONAHA.118.035273 30566022 PMC6296850

[B53] Pant T. Dhanasekaran A. Fang J. Bai X. Bosnjak Z. J. Liang M. (2018). Current status and strategies of long noncoding RNA research for diabetic cardiomyopathy. *BMC Cardiovasc. Disord.* 18:197. 10.1186/s12872-018-0939-5 30342478 PMC6196023

[B54] Porrello E. R. Mahmoud A. I. Simpson E. Hill J. A. Richardson J. A. Olson E. N. (2011). Transient regenerative potential of the neonatal mouse heart. *Science* 331 1078–1080. 10.1126/science.1200708 21350179 PMC3099478

[B55] Puente B. N. Kimura W. Muralidhar S. A. Moon J. Amatruda J. F. Phelps K. L. (2014). The oxygen-rich postnatal environment induces cardiomyocyte cell-cycle arrest through DNA damage response. *Cell* 157 565–579. 10.1016/j.cell.2014.03.032 24766806 PMC4104514

[B56] Rindler P. M. Crewe C. L. Fernandes J. Kinter M. Szweda L. I. (2013). Redox regulation of insulin sensitivity due to enhanced fatty acid utilization in the mitochondria. *Am. J. Physiol. Circ. Physiol.* 305 H634–H643. 10.1152/ajpheart.00799.2012 23792672

[B57] Schonrock N. Harvey R. P. Mattick J. S. (2012). Long noncoding RNAs in cardiac development and pathophysiology. *Circ. Res.* 111 1349–1362. 10.1161/CIRCRESAHA.112.268953 23104877

[B58] Song K. Nam Y. J. Luo X. Qi X. Tan W. Huang G. N. (2012). Heart repair by reprogramming non-myocytes with cardiac transcription factors. *Nature* 485 599–604. 10.1038/nature11139 22660318 PMC3367390

[B59] Soonpaa M. H. Kim K. K. Pajak L. Franklin M. Field L. J. (1996). Cardiomyocyte DNA synthesis and binucleation during murine development. *Am. J. Physiol.* 271 H2183–H2189. 10.1152/ajpheart.1996.271.5.H2183 8945939

[B60] Stein C. S. Jadiya P. Zhang X. McLendon J. M. Abouassaly G. M. Witmer N. H. (2018). Mitoregulin: a lncRNA-Encoded microprotein that supports mitochondrial supercomplexes and respiratory efficiency. *Cell Rep.* 23 3710.e8–3720.e8. 10.1016/j.celrep.2018.06.002 29949756 PMC6091870

[B61] Thum T. Condorelli G. (2015). Long noncoding RNAs and microRNAs in cardiovascular pathophysiology. *Circ. Res.* 116 751–762. 10.1161/CIRCRESAHA.116.303549 25677521

[B62] Tzahor E. Poss K. D. (2017). Cardiac regeneration strategies: staying young at heart. *Science* 356 1035–1039. 10.1126/science.aam5894 28596337 PMC5614484

[B63] Uygur A. Lee R. T. (2016). Mechanisms of cardiac regeneration. *Dev. Cell* 36 362–374. 10.1016/j.devcel.2016.01.018 26906733 PMC4768311

[B64] van Gastel N. Stegen S. Eelen G. Schoors S. Carlier A. Daniëls V. W. (2020). Lipid availability determines fate of skeletal progenitor cells via SOX9. *Nature* 579 111–117. 10.1038/s41586-020-2050-1 32103177 PMC7060079

[B65] van Soligen C. (2018). Long noncoding RNAs in lipid metabolism. *Curr. Opin. Lipidol.* 29 224–232. 29553997 10.1097/MOL.0000000000000503 PMC6077844

[B66] Vausort M. Wagner D. R. Devaux Y. (2014). Long noncoding RNAs in patients with acute myocardial infarction. *Circ. Res.* 115 668–677. 10.1161/CIRCRESAHA.115.303836 25035150

[B67] Viereck J. Bührke A. Foinquinos A. Chatterjee S. Kleeberger J. A. Xiao K. (2020). Targeting muscle-enriched long non-coding RNA H19 reverses pathological cardiac hypertrophy. *Eur. Heart J.* 41 3462–3474. 10.1093/eurheartj/ehaa519 32657324 PMC8482849

[B68] Vivien C. J. Hudson J. E. Porrello E. R. (2016). Evolution, comparative biology and ontogeny of vertebrate heart regeneration. *npj Regen*. *Med.* 1:16012. 10.1038/npjregenmed.2016.12 29302337 PMC5744704

[B69] Wang K. Long B. Zhou L.-Y. Liu F. Zhou Q.-Y. Liu C.-Y. (2014). CARL lncRNA inhibits anoxia-induced mitochondrial fission and apoptosis in cardiomyocytes by impairing miR-539-dependent PHB2 downregulation. *Nat. Commun.* 5:3596. 10.1038/ncomms4596 24710105

[B70] Wang S. Zuo H. Jin J. Lv W. Xu Z. Fan Y. (2019). Long noncoding RNA Neat1 modulates myogenesis by recruiting Ezh2. *Cell Death Dis.* 10:505. 10.1038/s41419-019-1742-7 31243262 PMC6594961

[B71] Wang Y. Hu S. B. Wang M. R. Yao R. W. Wu D. Yang L. (2018). Genome-wide screening of NEAT1 regulators reveals cross-regulation between paraspeckles and mitochondria. *Nat. Cell Biol.* 20 1145–1158. 10.1038/s41556-018-0204-2 30250064

[B72] Yan C. Chen J. Chen N. (2016). Long noncoding RNA MALAT1 promotes hepatic steatosis and insulin resistance by increasing nuclear SREBP-1c protein stability. *Sci. Rep.* 6:22640. 10.1038/srep22640 26935028 PMC4776244

[B73] Yang F. Zhang H. Mei Y. Wu M. (2014). Reciprocal regulation of HIF-1α and lincRNA-p21 modulates the Warburg effect. *Mol. Cell* 53 88–100. 10.1016/j.molcel.2013.11.004 24316222

[B74] Yucel N. Wang Y. X. Mai T. Porpiglia E. Lund P. J. Markov G. (2019). Glucose metabolism drives histone acetylation landscape transitions that dictate muscle stem cell function. *Cell Rep.* 27 3939.e6–3955.e6. 10.1016/j.celrep.2019.05.092 31242425 PMC6788807

[B75] Zhang L. Zhou Y. Huang T. Cheng A. S. L. Yu J. Kang W. (2017). The interplay of LncRNA-H19 and its binding partners in physiological process and gastric carcinogenesis. *Int. J. Mol. Sci.* 18:450. 10.3390/ijms18020450 28230721 PMC5343984

[B76] Zhang M. Chi X. Qu N. Wang C. (2018). Long noncoding RNA lncARSR promotes hepatic lipogenesis via Akt/SREBP-1c pathway and contributes to the pathogenesis of nonalcoholic steatohepatitis. *Biochem. Biophys. Res. Commun.* 499 66–70. 10.1016/j.bbrc.2018.03.127 29555473

[B77] Zhao X.-Y. (2015). Long noncoding RNAs: a new regulatory code in metabolic control. *Trends Biochem. Sci.* 40 586–596. 26410599 10.1016/j.tibs.2015.08.002 PMC4584418

[B78] Zhuo C. Jiang R. Lin X. Shao M. (2017). LncRNA H19 inhibits autophagy by epigenetically silencing of DIRAS3 in diabetic cardiomyopathy. *Oncotarget* 8 1429–1437. 10.18632/oncotarget.13637 27903964 PMC5352066

[B79] Zolk O. Solbach T. F. Eschenhagen T. Weidemann A. Fromm M. F. (2008). Activation of negative regulators of the hypoxia-inducible factor (HIF) pathway in human end-stage heart failure. *Biochem. Biophys. Res. Commun.* 376 315–320. 10.1016/j.bbrc.2008.08.152 18782560

